# GERD-related chronic cough: Possible mechanism, diagnosis and treatment

**DOI:** 10.3389/fphys.2022.1005404

**Published:** 2022-10-20

**Authors:** Jiankang Wu, Yiming Ma, Yan Chen

**Affiliations:** Department of Pulmonary and Critical Care Medicine, The Second Xiangya Hospital, Central South University, Changsha, Hunan, China

**Keywords:** gastroesophageal reflux disease, chronic cough, mechanism, diagnosis, treatment

## Abstract

GERD, or gastroesophageal reflux disease, is a prevalent medical condition that affects millions of individuals throughout the world. Chronic cough is often caused by GERD, and chronic cough caused by GER is defined as GERD-related chronic cough (GERC). It is still unclear what the underlying molecular mechanism behind GERC is. Reflux theory, reflex theory, airway allergies, and the novel mechanism of esophageal motility disorders are all assumed to be linked to GERC. Multichannel intraluminal impedance combined with pH monitoring remains the gold standard for the diagnosis of GERC, but is not well tolerated by patients due to its invasive nature. Recent discoveries of new impedance markers and new techniques (mucosal impedance testing, salivary pepsin, real-time MRI and narrow band imaging) show promises in the diagnosis of GERD, but the role in GERC needs further investigation. Advances in pharmacological treatment include potassium-competitive acid blockers and neuromodulators (such as Baclofen and Gabapentin), prokinetics and herbal medicines, as well as non-pharmacological treatments (such as lifestyle changes and respiratory exercises). More options have been provided for the treatment of GERC other than acid suppression therapy and anti-reflux surgery. In this review, we attempt to review recent advances in GERC mechanism, diagnosis, and subsequent treatment options, so as to provide guidance for management of GERC.

## 1 Introduction

Cough that lasts more than 8 weeks is described as chronic cough, which is a frequent clinical condition that significantly reduces one’s quality of life (French et al., 1998; Jin and Kim, 2020). The incidence of chronic cough is estimated to range from 9% to 33% in Europe and the United States (Chung and Pavord, 2008), leading to a significant socioeconomic burden (Francis et al., 2013). As for the etiology of chronic cough, when other causes (such as asthma and postnasal drip) have been ruled out, GERD should be considered (Rodriguez-Tellez, 2005). Approximately 10–59% of chronic cough cases are caused by GERD (Chang et al., 2011). GERD, as defined by the Montreal Consensus, is the reflux of gastrointestinal contents that causes symptoms or complications (Vakil et al., 2006). The recent Lyon consensus on GERD considers that symptoms may be unreliable and recommends that it should be diagnosed only with specific endoscopic findings (severe esophagitis, intestinal metaplasia or stricture of long segments of esophageal mucosa) or an esophageal acid exposure time greater than 6% of the 24-h pH monitoring study time (Gyawali et al., 2018). Chronic cough and persistent dysphonia are the main complaints of extraesophageal manifestations of GERD (Ahmad and Batch, 2004; Belafsky et al., 2008). As opposed to western countries, it is interesting to note that previously the cause of chronic cough in Asians was rarely attributed to GERD (El-Serag et al., 2014), chronic cough associated with it have increased as well due to an increase in the prevalence of GERD in Japan (Niimi, 2017) and in China (Ding et al., 2019). All in all, The most common cause of chronic cough is GERD, and chronic cough caused by GERD is a subtype of GERD-related disease (Kahrilas et al., 2016; Ding et al., 2019), which is known as GERD-related chronic cough (GERC). In this review, we discuss recent advances in the diagnosis and treatment of GERC as well as their mechanisms.

## 2 Mechanism

**FIGURE 1:**
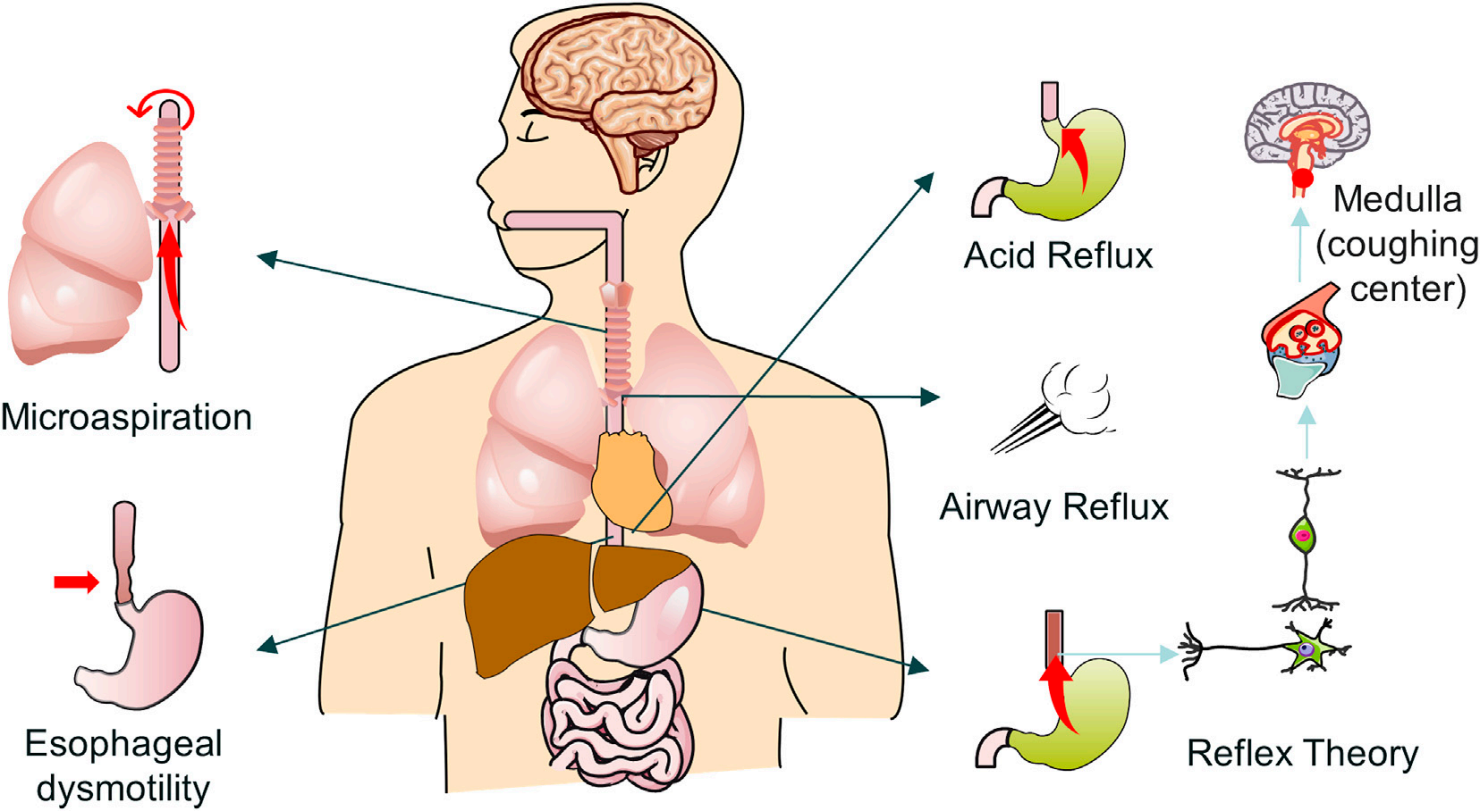
Possible mechanism of GERC.

There are three possible pathophysiological mechanisms that contribute to the development of GERC (Figure 1). The first is the “reflux theory”, which includes acid reflux, microaspiration and airway reflux; the second is the “reflex theory”, which includes acid reflex and weak acid reflex; and the third is “esophageal dysmotility” (Xu et al., 2015).

### 2.1 Reflux theory

The reflux theory, also known as proximal reflux or micro/major aspiration theory, indicates that the structural and functional abnormalities of the lower esophagus lead to the reflux of gastric contents. Reflux typically involves four mechanisms: transient lower esophageal sphincter relaxations (TLESRs), low LES pressure, swallowing-related LES relaxation, and tension during periods of low LES pressure. Regarding TLESRs, defined by conventional manometry as (A) no pharyngeal swallowing signal from 4 s before the onset of LES relaxation to 2 s after the onset of relaxation, (B) LES pressure reduction ≥1 mm Hg/s, (C) time from onset to complete relaxation ≤10 s, and (D) minimum pressure ≤2 mm Hg, have been considered the most important pathogenic mechanisms in patients with GERD (Holloway et al., 1995; Mittal et al., 1995; Ribolsi et al., 2020). Mechanisms to prevent reflux vary depending on the physiological setting and the anatomy of the esophagogastric junction (EGJ). For example, the diaphragm may play a major protective role in the presence of a sudden increase in intra-abdominal pressure and tension. In contrast, basal LES pressure may play a major protective role in the resting recumbent and postprandial states, and low LES pressure may predispose patients to more reflux at night and after meals (Tack and Pandolfino, 2018). Reflux can stimulate cough receptors directly, or it can cause mucus to be secreted in the lower respiratory tract through the vagal reflex and activate cough receptors (Patterson et al., 2009).

### 2.2 Acid reflux

There is strong evidence that acid reflux is one of the leading causes of chronic cough, while some patients with chronic cough have shown significant improvement in their symptoms after receiving antacid therapy (Park et al., 2017; Yu et al., 2017). Anti-acid therapy, however, is not effective for many patients. A study found that weak acid reflux contributes to GERC (Mainie et al., 2005; Sifrim et al., 2005; Ghezzi et al., 2013). It was found that a large amount of non-acidic reflux was present in the proximal esophagus and larynx of patients with non-acidic GERC, which accounted for 73% and 11% of the total reflux, respectively. Moreover, reflux activated cough receptors that had previously been activated by the vagal reflex. Massive reflux thickens the lower esophagus, causing structural and functional abnormalities, as well as causing reflux into the upper esophagus. Therefore, some patients with non-acid GERC have a cough that can be explained by reflux theory (Oelschlager et al., 2006; Patterson et al., 2009).

A multicentre study indicates that volumetric clearance time, reflux burden, and proximal extent are all relevant considerations in the development of GERC; The presence of prolonged massive reflux appears to be the main cause of sudden coughing, and ph and clearance times do not seem to matter. This explains findings that acid suppression therapy does not help certain patients with chronic cough (Herregods et al., 2017).

### 2.3 Microaspiration

Additionally, stomach contents have been demonstrated in some cases to enter the lungs and cause aspiration inflammation (Ravelli et al., 2006). A hypothesis regarding the mechanism of GERC is that microaspiration of proximal reflux and stomach reflux stimulates coughing through direct irritation of the respiratory tract. Reduced sensitivity of protective reflexes in the larynx and pharynx, decreased swallowing coordination, and esophageal motility abnormalities are thought to contribute to the risk of microaspiration (Phua et al., 2005). GERC can be triggered by proximal acid reflux and aspiration of stomach contents (pepsin, bile acid, or lipidloded macrophages), according to previous research (Farrell et al., 2006; Grabowski et al., 2011; Ozdemir et al., 2017). However, other investigations have revealed no changes in proximal reflux episodes, bronchoalveolar lavage (BAL) pepsin, or bile acids between chronic cough patients and controls (Sifrim et al., 2005; Decalmer et al., 2012). Thus, the involvement of proximal gastric reflux and airway microaspiration in chronic cough is restricted. Furthermore, cough appears to protect people with persistent cough by lowering the levels of pepsin in the respiratory tract (Decalmer et al., 2012).

### 2.4 Airway reflux

Over time, the concept of airway reflux, which consists primarily of a gaseous mist of non-acidic composition, has gained widespread acceptance. The 24-h pH-impedance method is the current gold standard for diagnosing GERD. Each reflux episode’s type, number, phase period, duration, and proximal extent are measured using pH-impedance (Castell and Vela, 2001; Sifrim et al., 2001; Kahrilas and Sifrim, 2008). However, no definitive investigations on the occurrence of airway reflux in GERC have been conducted to far. The role of airway reflux in GERC can be studied in the future using pH impedance techniques.

### 2.5 Reflex theory

Another proposed explanation for GERC is the distal esophageal afferent nerve-mediated esophagus-tracheobronchial reflex, also known as the reflex theory. GERC is associated with increased sensitivity of the cough reflex and the development of neurogenic airway inflammation (Smith et al., 2010; Qiu et al., 2011; Kahrilas et al., 2016). The reflex theory, also known as the esophago-tracheo-bronchial reflex theory, proposes that stimulation of subesophageal mucosal receptors by reflux substances activates the cough center through the esophagus and ultimately causes the bronchial cough reflex. Through cytoplasmic division, the matching efferent nerve terminals release neuropeptides such as substance P at the same moment. These neuropeptides can cause neurogenic inflammation or activate neuropeptide receptors on mast cell surfaces, releasing inflammatory mediators such as trypsin, histamine, and prostaglandin E2, which ultimately stimulate cough receptors and lead to cough (Niimi et al., 2005). In tests comparing saline and acid infusions, researchers discovered that acid causes cough more frequently and to a higher extent than saline, and that esophageal infusion of acid increases cough sensitivity in patients with GERD and cough (Ing et al., 1994; Javorkova et al., 2008). Furthermore, patients with unexplained cough had reduced symptoms of cough and distal reflux after anti-GERD treatment, which helps to promote the mechanism of the distal-reflux reflex (Hu et al., 2014).

Besides, it has been shown that non-acidic esophageal reflux passing through Aδ fibers activates mechanical stretch receptors to cause cough, whereas acidic reflux activates chemoreceptors transient receptor potential vanilloid type 1 (TRPV1) to stimulate the vagus nerve and cause cough *via* the esophago-tracheo-bronchial reflex. Studies suggest that chronic cough caused by non-acid reflux may be associated with neurogenic airway inflammation and cough reflex hypersensitivity caused by mast cell activation, of which weak acid reflux may be the main factor (Qiu et al., 2011). These results show the reflex theory that a weak acid-induced esophagogastric-tracheobronchial reflex is crucial in nonacid GERC.

### 2.6 Esophageal dysmotility

Another key GERD mechanism is esophageal dysmotility. According to studies, GERC episodes are linked to ineffective esophageal motility (Fouad et al., 1999; Knight et al., 2000) and massive esophageal ruptures (Almansa et al., 2015; Bennett et al., 2018). Moreover, esophageal body motility is inversely linked with prolonged acid exposure (Jiang et al., 2017; Jiang et al., 2019). The majority of primary and secondary esophageal peristalsis were ineffective (61.8%–88.6%); Both distal and proximal esophageal low-pressure amplitudes resulted in 38.5% IEM (Ineffective esophageal motility) in primary esophageal peristalsis, IEM is defined as the presence of peristaltic waves of amplitude <30 mmHg and/or non-transmissive contractions in the distal esophagus (Spechler and Castell, 2001), and IEM occurs in GERC patients as a result of increased synchronized contractions of esophageal peristalsis. In addition, Diener et al. showed that GERD patients with IEM have longer esophageal acid exposure, slower clearance, more frequent reflux episodes as well as more severe mucosal damage and more frequent respiratory symptoms (Diener et al., 2001). These findings support the hypothesis that primary and secondary intestinal peristalsis are compromised, resulting in poor esophageal clearance and extended acid exposure (Aben-Athar and Dantas, 2006; Iwakiri et al., 2007). Therefore, it can be infered that the low whole esophageal pressure amplitude during primary gastric peristalsis and the synchronous contraction during secondary gastric peristalsis play a significant impact in GERC (Li et al., 2019).

## 3 Risk factors

Several GERC-related risk factors are discussed below that may be helpful in the management and treatment of GERC.

### 3.1 Obesity

Obesity is a major risk factor for GERD symptoms, with a ratio of 1.73 (Eusebi et al., 2018). Observational studies have shown that reducing body mass index by at least 3.5 kg/m2 increases the odds of resolving GERD symptoms by a factor of 1.5–2.4. In addition, randomized trials have confirmed that weight loss, especially waist circumference reduction, improves GERD symptoms and reduces acid exposure in the esophagus (Ness-Jensen et al., 2016). It is therefore reasonable to conclude that the obese population may be more susceptible to GERC.

### 3.2 Smoking

This is supported by an 18-year longitudinal study in which people who reduced smoking were three times more likely to have acid reflux and heartburn symptoms than those who continued to smoke. Tobacco is also a significant risk factor for corrosive esophagitis and esophageal adenocarcinoma (Lewis et al., 2011; Francis et al., 2015). So the effects of smoking on the respiratory tract may cause patients to be more likely to develop cough.

### 3.3 Drinking alcohol

Patients with GERD symptoms often report that drinking alcohol worsens their symptoms, and randomized studies have demonstrated that ingesting alcohol is more likely to trigger acid reflux than water (Pehl et al., 2002; Pehl et al., 2006). Therefore, it can be concluded that people who drink alcohol regularly may be more likely to develop cough when suffering from GERD.

### 3.4 Physical activity

The relationship between physical activity and GERD is also complex. On the one hand, certain forms of physical activity are associated with an increased risk of GERD. For example, bending, bicycling, weight lifting, swimming, and even surfing have been associated with an increased risk of GERD, especially during or shortly after activity (Collings et al., 2003; Norisue et al., 2009). It may also contribute to the development of GERC.

## 4 Diagnosis

**FIGURE 2:**
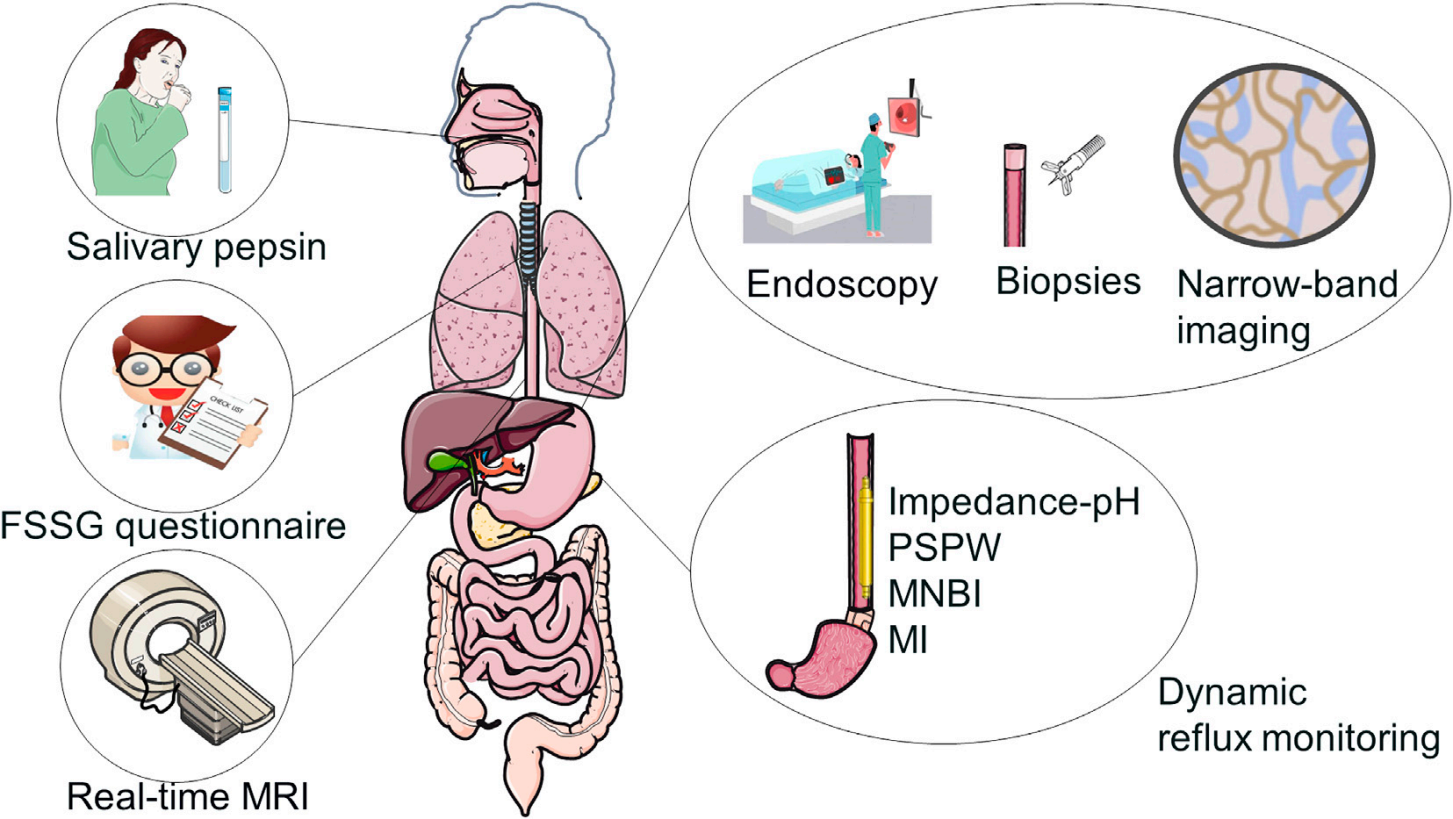
Diagnostic tests of GERC.

When patients have no signs of reflux, it might be difficult to identify GERC. According to reports, about 70% of GERC patients do not have typical gastrointestinal reflux symptoms such heartburn or acid reflux (Laukka et al., 1994; Kahrilas et al., 2016). As a result, the American College of Chest Physicians (ACCP) recommends excluding other conditions that cause chronic cough when predicting GERC (Kahrilas et al., 2016). Therefore, the diagnosis of GERC requires consideration of the history of cough and reflux, as well as the identification of alternative causes of persistent cough. Below are some specific tests that can help diagnose GERC (Figure 2), as well as a table and flow chart of the advantages and disadvantages of different diagnostic methods (Supplementary Table S1, Supplementary Figure S1).

### 4.1 Endoscopy and biopsies

Endoscopy is the preferred screening method for suspected GERD syndrome due to its diagnostic efficiency, relative safety, biopsy capabilities, therapeutic promise, and relative specificity (Committee et al., 2015; Mann et al., 2021). However, so far, endoscopy has not shown great advantages in the detection of GERC due to its low diagnostic rate and low patient tolerance.

The function of biopsy in the diagnosis of adult GERD has yet to be determined. The disadvantages are time-consuming, expensive, and less sensitive, whereas the advantages are that it can be helpful when the predicted risk of GERD is high and endoscopy cannot show a significant positive result (Vakil et al., 2017). The site of the esophageal biopsy may be crucial, because abnormalities are more likely to occur in the esophageal quadrant following the lesser curvature of the stomach (Edebo et al., 2007). In theory, a simple, repeatable, and sensitive biomarker that does not necessitate specialist pathology testing or costly procedures could be very useful in clinical practice.

According to recent research, a total distal esophageal epithelial thickness of at least 430 mm may be a marker for GERD (Vieth et al., 2016). Using a total distal esophageal epithelial thickness of at least 430 mm as a criterion (together with endoscopic, pH monitoring-based criteria for investigation of gastrointestinal disease), the diagnostic yield of biopsied patients increased by 12% (from 55% to 67%) (Vakil et al., 2017). In GERC patients with predominantly cough symptoms, measurement of epithelial thickness may decrease additional costs, discomfort and personal inconvenience of esophageal pH monitoring, and this still awaits further studies.

### 4.2 Dynamic reflux monitoring

#### 4.2.1 Impedance pH detection

Impedance-pH monitoring is often regarded as the most precise and thorough tool for evaluating GERD and has greatly improved the diagnosis of GERD patients. Recent studies have shown it to be an effective tool for measuring acidic and non-acidic reflux, as well as an improved way to assess the symptoms of GERD (Sifrim et al., 2004). Detects whether the reflux content is liquid, gaseous or mixed by ph-impedance measurements without regard to acidity. When a liquid travels across an electrode, for example, the impedance is reduced due to the low resistance of the liquid. Furthermore, the low ionic density of the air causes a high resistance to electrical currents and a high impedance to gas flow during hiccups. A pH impedance catheter probe with an impedance electrode uses this knowledge to analyze the direction of pill movement along the esophagus lumen and to record the frequency of prograde motion after swallowing or retrograde motion during reflux (Prakash and Jonnalagadda, 2006). It is particularly useful when transnasal catheters are not tolerated or when there is high suspicion of a negative result in the previous diagnosis of GERD (Sweis et al., 2011; Penagini et al., 2015). However, wireless pH monitoring is expensive, limiting its availability. Also the increased diagnostic rate is limited and ancient this test is not widely available (Savarino et al., 2011).

Esophageal pH monitoring combined with impedance method is useful to distinguish the type of reflux, which can be classified as acid reflux, weak acid reflux and alkaline reflux. Acid reflux is defined as a reflux event with an esophageal pH of less than 4, weak acid reflux is defined as a pH of 4–7, and alkaline reflux is defined as a pH of greater than or equal to 7 (Gyawali et al., 2020). Several studies have demonstrated that increased esophageal impedance monitoring is crucial for identifying reflux as a cause of symptoms (Bredenoord et al., 2006; Zerbib et al., 2006; Hemmink et al., 2008).

In GERC cases, esophageal pH monitoring in patients is a sensitive method for diagnosis. It is also crucial to integrate 24-h pH impedance monitoring with objective cough episode detection. For example, 24-h dynamic manometry is performed simultaneously so that the temporal correlation between all types of reflux episodes (acidic and weakly acidic) and all cough episodes can be assessed. There was a link between cough and weakly acidic reflux in patients with chronic cough of uncertain origin. Impedance-pH-pressometry identifies patients whose cough may be associated with reflux, as well as patients whose coughs were overlooked by standard reflux diagnostic criteria (Blondeau et al., 2007; Bogte et al., 2008; Roman et al., 2017; Herregods et al., 2018).

#### 4.2.2 Symptom index (SI) and symptom association probability (SAP)

In addition to quantifying esophageal acid or non-acid exposure, The relationship between reported symptoms and acid or non-acid reflux occurrences can be measured *via* pH and impedance monitoring. The two most widely utilized metrics are the symptom index (SI) and symptom association probability (SAP) (Weusten et al., 1994). During the trial period, SI indicate the percentage of reflux-related symptom events. However, this method omits the total number of reflux events from the calculation.

The likelihood of a positive SI was later discovered to rise with the frequency of reflux episodes. The symptom association probability (SAP), a complicated statistical method for detecting whether reflux patterns and symptoms occur by chance, was developed as a result of this (Weusten et al., 1994).

The SI and SAP interpretations are complementary, with the SI indicating the relationship’s effect size and the SAP indicating the relationship’s likelihood (Gyawali et al., 2018). There is substantial evidence of a link between reflux and symptoms when the two values show a positive association. The fact that the two tests are frequently one positive and one negative, however. Other characteristics such as the duration of acid exposure, the frequency of reflux episodes, and clinical symptoms, according to the Porto Consensus, should be considered when determining the reflux-symptom connection (Roman et al., 2017). Furthermore, because SAP is a probabilistic calculation, it is seen to have more statistical validity in clinical practice and, as a result, by some to be a superior assessment of symptoms (Kushnir et al., 2012; Gyawali et al., 2020). In addition, some studies have shown that SAP is a more sensitive diagnostic method than SI for GERC (Rybka et al., 2014).

### 4.3 Other impedance parameters

#### 4.3.1 Mean nocturnal baseline impedance (MNBI)

An alternative impedance marker is MNBI, which uses impedance values during sleep and is preferable to daytime values that are susceptible to daytime swallowing (Martinucci et al., 2014). Studies have shown that lower MNBI values help distinguish between different types of esophagitis, cough and NERD (non-erosive reflux disease) as well as functional heartburn and GERD/NERD in healthy controls (de Bortoli et al., 2015; Frazzoni et al., 2016; Frazzoni et al., 2017a). Impaired esophageal mucosal integrity, as evidenced by a low MNBI, has recently been found to boost the diagnostic yield of impedance-pH in indeterminate GERD patients and can be combined with AET (acid exposure time) to determine if an individual is responding to anti-reflux treatment (Rengarajan et al., 2020).

#### 4.3.2 DeMeester score

The DeMeester score (DMS) is a six-parameter composite score used to assess acid exposure during long-term dynamic pH monitoring, such as acid exposure time (AET). A study has shown that both AET and DMS are equally accurate in detecting GERC. If AET reaches 4.8%, then GERC is suspected, whereas acid GERC is suspected if AET reaches 6.2% (Neto et al., 2019; Zhu et al., 2021a).

#### 4.3.3 Post-reflux swallow-induced peristaltic wave (PSPW)

The prograde course of the impedance drop within 30 s following a reflux episode is called the PSPW in pH-impedance studies, and it is lower in GERD than in controls. Divide the number of PSPW by the proportion of reflux episodes to get the PSPW index (de Bortoli et al., 2015; Frazzoni et al., 2016; Frazzoni et al., 2017b). The PSPW index allows assessment of the integrity of reflux-stimulated primary peristalsis and is associated with multiple contractile reserve assessed by rapid swallowing. In addition, recent research using the PSPW index to investigate esophageal chemical clearance mechanisms have found lower chemical clearance values in GERD patients compared to controls. When other impedance-pH parameters are unknown, this variable might be used as a diagnostic tool (Frazzoni et al., 2013; Frazzoni et al., 2016).

#### 4.3.4 Mucosal impedance (MI)

An important clinical observation is that in patients with severe reflux disease, analysis of dynamic impedance measurements is inaccurate due to the low baseline values which are difficult to interpret. This finding implies that GERD may cause chronic changes in the esophageal epithelium and that impedance is difficult to detect chronic changes over time. This concept facilitated the creation of MI, which involves the placement of a probe under the endoscope that passes through the working channel of the endoscope and directly contacts the mucosa thereby measuring impedance (Ates et al., 2015).

Baseline impedance is highly sensitive in detecting GERC and distinguishing between different phenotypes, with a reported specificity of 95% and a positive predictive value of 96% (Naik et al., 2019). Low baseline impedance has been associated with LES insufficiency, esophageal hypersensitivity, abnormal esophageal acid exposure, typical GERD symptoms, and positive acid perfusion tests (Woodland et al., 2013; Zhong et al., 2013; Arul et al., 2015; Lottrup et al., 2017). The role of MI in GERC needs further investigation.

#### 4.3.5 Real-time magnetic resonance imaging (MRI)

A study proposes a non-invasive real-time MRI method to evaluate GERD. Modern ultrafast MRI sequences visualize the esophageal and gastroesophageal junction and allow dynamic assessment of reflux during repeated Valsalva manipulations with high tissue contrast of the surrounding anatomy and temporal resolution up to 20 ms (Olthoff et al., 2014; Zhang et al., 2014). In those who are presenting GERD symptoms, the diagnostic performance of real-time MRI for GERD detection is superior to that of invasive pH measurements and impedance. Images on real-time MRI can show either acidic or non-acidic reflux and can identify patients with non-acidic reflux that is not detected by other methods. To date, there is insufficient evidence that real-time MRI can replace existing diagnostic methods, but instead serve as an adjunctive diagnostic tool, particularly for individuals who are intolerant of 24-h esophagus and for identifying candidates for anti-reflux surgery (Seif Amir Hosseini et al., 2020).

#### 4.3.6 Proton pump inhibitor trial

This is a practical approach, but response to PPI therapy is not equivalent to the diagnosis of GERD, especially since GERD has many atypical extraesophageal symptoms. For example, the response rate to PPI for chest pain, chronic cough, and pharyngitis is much lower than that of heartburn patients, thus reducing the utility of this diagnostic method (Gyawali and Fass, 2018). In addition, its main limitation is how to select suitable patients for the trial. Despite the low specificity, high placebo response (Numans et al., 2004) and the low cost of empirical PPI treatment compared with diagnostic tests (Jonasson et al., 2012), it undoubtedly leads to overdiagnosis and overuse of PPI.

#### 4.3.7 Narrow-band imaging

Narrow band imaging (NBI) uses spectral narrow band filters to display mucosal patterns and microvasculature, enhancing contrast and allowing detection of changes in mucosal microvasculature in patients with GERD, enabling identification of small changes such as increased vascularity at the villous mucosal surface, mucosal islands, microerosions and squamous columnar junctions (Lynch et al., 2013). Therefore, NBI is used to detect gastroesophageal reflux and monitor its improvement following treatment with Proton-pump inhibitor (PPI). NBI detected tiny foci of inflammation in the esophagus in 82 individuals, which corresponded with a favourable response to PPI treatment (Tseng et al., 2011). Although NBI is not the first-line method for diagnosing GERD, it can be used as an adjunct to diagnosis and response to PPI therapy due to the cost of endoscopy.

#### 4.3.8 Salivary pepsin

Pepsin, formed by the activation of pepsinogen released from gastric principal cells, is a protein hydrolase. Salivary pepsin assay is considered to be a promising non-invasive method for detecting gastroesophageal reflux (Potluri et al., 2003). There are now two main methods available: immunological and enzymatic assays. Enzymatic analysis is difficult to obtain, standardized, and has a tendency to lose sensitivity, hence immunological analysis is preferable (Samuels and Johnston, 2010). Considering the non-invasive and simple nature, this test would be suitable for diagnosing GERD or extraesophageal reflux like GERC in children, compared to pH measurements or pH impedance monitoring which compare well. Unfortunately, the current data are inconclusive or negative (Ocak et al., 2015; Yadlapati et al., 2016). For example, despite the fact that salivary pepsin has been proven in some studies to aid in the diagnosis of laryngopharyngeal reflux (Hayat et al., 2014; Sereg-Bahar et al., 2015; Spyridoulias et al., 2015), recent studies have failed to demonstrate the test’s clinical utility (Yadlapati et al., 2016; Race et al., 2019). Defining the optimal saliva collection protocol, or detecting pepsin by other means such as sputum, may aid in the development of this test as a viable GERC detection tool.

#### 4.3.9 Frequency of symptoms of gastroesophageal reflux scale (FSSG)

The FSSG is a validated symptom questionnaire for esophagogastroesophagitis consisting of 12 primary symptoms (7 with acid-related symptoms and 5 with dyskinesia symptoms) and two additional dyskinesia symptoms, the latter two newly combined with dyspeptic symptoms. When the score threshold is 7, it is a valuable diagnostic tool in people with subacute/chronic cough. When GERC patients have a blood eosinophil level of 150/µL or fewer, studies have suggested that FSSG may be more beneficial (Kusano et al., 2004; Kurokawa et al., 2021).

## 5 Treatment

**FIGURE 3:**
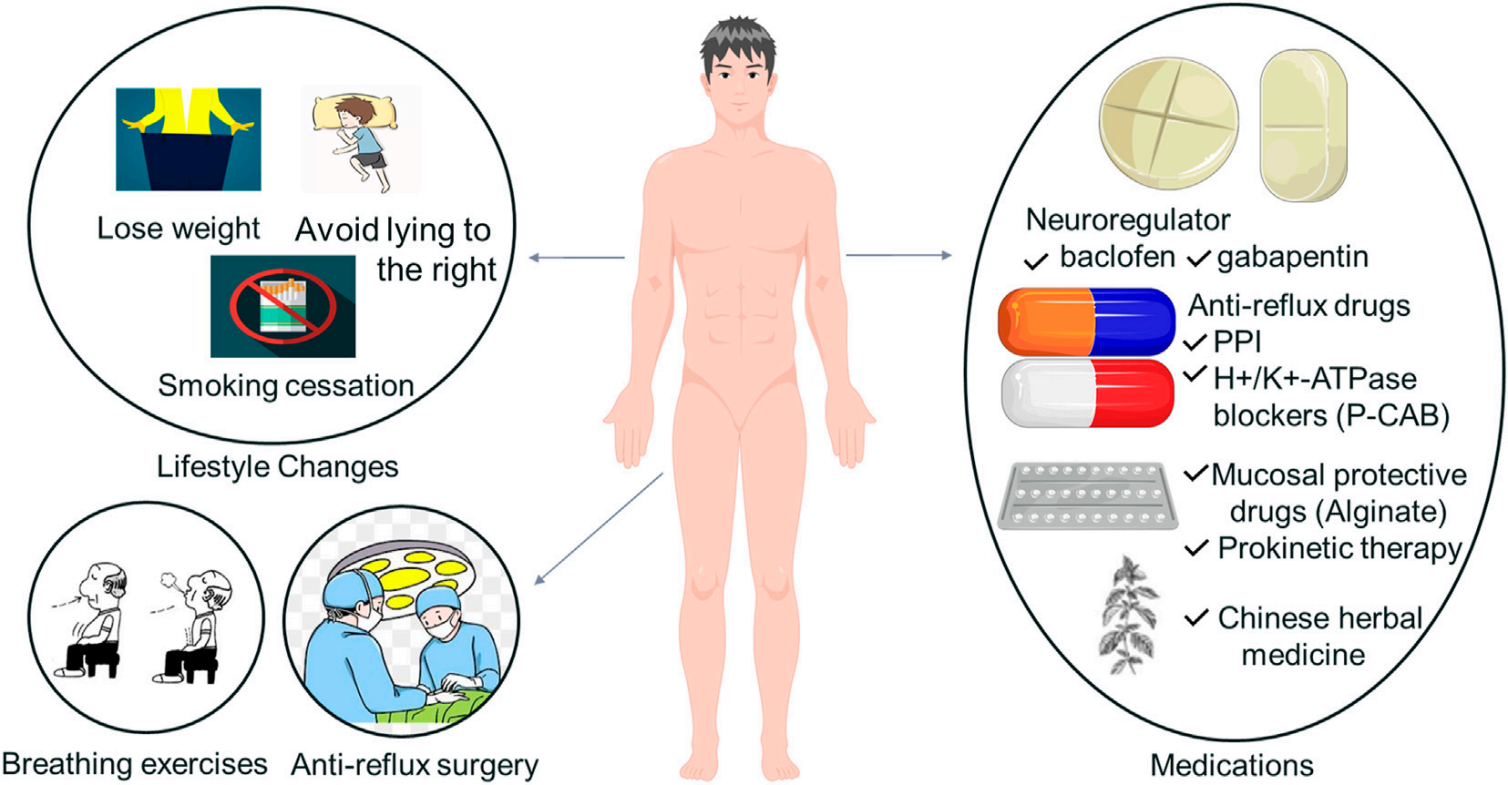
Specific treatments of GERC.

Today the treatment regarding GERC is still based on the treatment of GERD. The main treatment methods are lifestyle changes, medications, anti-reflux surgery and the latest methods to be further observed, the specific treatment measures and their application and prospects in GERC are mainly listed below (Figure 3). In addition we also provide tables and flow charts on treatment mechanisms and advantages and disadvantages (Supplementary Table S2, Supplementary Figure S2).

### 5.1 Lifestyle changes

Lifestyle changes are an important part of the treatment of patients with reflux symptoms. Despite the fact that a recent study of patients with extraesophageal reflux found that lifestyle changes alone were less effective than PPIs plus lifestyle changes (Chappity et al., 2014). In a study of 223 patients with extraesophageal symptoms thought to be related to reflux, researchers discovered a substantial nonlinear relationship between esophageal acid exposure and body mass index (BMI) (Aslam et al., 2012). More research is needed into the role of weight loss in treating or lowering extraesophageal symptoms. One study suggests that lying to the right increases nocturnal reflux and postprandial reflux and recommends that patients avoid sleeping lying to the right (Khan et al., 2012; Person et al., 2015; Allampati et al., 2017). Another large cohort study showed that quitting smoking improved GERD symptoms (Ness-Jensen et al., 2014). And further studies showed a 44% improvement in GERC symptoms in patients who successfully quit smoking for 1 year, compared to 18% in those who continued smoking (Kohata et al., 2016). It is well known that smoking cessation is one of the important treatments for many lung diseases, and the effect of smoking cessation on GERC awaits further studies.

### 5.2 Medications

#### 5.2.1 Baclofen

Baclofen is an agonist of the γ-aminobutyric acid type-B receptor. It is been used to treat spastic muscular diseases in the past, but it is also been utilized to treat refractory gastric reflux disease (Sifrim and Zerbib, 2012; Li et al., 2014). Its main principle is to modulate transient LES relaxation mediated by the vagal reflex pathway through activation of GABA type-B receptors (Zhang et al., 2002).

In addition to inhibiting TLESRs (transient lower esophageal sphincter relaxation), baclofen has non-specific cough suppressant activity in humans (Dicpinigaitis and Dobkin, 1997; Dicpinigaitis et al., 1998). Some evidence suggests that baclofen is helpful in angiotensin-converting enzyme inhibitor-induced cough (Dicpinigaitis, 1996), and unexplained chronic refractory cough (Dicpinigaitis and Rauf, 1998). Therefore baclofen was studied in an intensive anti-reflux regimen as an add-on therapy to an antisecretory drug for suspected refractory GERC. Cough relief after baclofen treatment was first demonstrated in three patients with GERC in a case report (Xu et al., 2012). The study found good results with baclofen in 38 patients resistant to high-dose anti-reflux drugs and significant symptom improvement in about 37 of 103 patients with suspected refractory GERC (Xu et al., 2016). As a result, baclofen may be an effective therapy option for refractory GERC.

Unfortunately, one study indicated that baclofen treatment was ineffective in 40% of individuals with refractory GERC (Dong et al., 2019). Baclofen reduces but does not eliminate TLESRs, so residual reflux may continue to cause coughing by stimulating receptors in the esophageal mucosa (Lv and Qiu, 2015). As a result, the therapeutic relevance and significance of screening the most suited individuals with refractory GERC for baclofen treatment is significant. A recent study suggests that LESP (lower esophageal sphincter pressure) and LESL (lower esophageal sphincter pressure) can be used to screen refractory GERC patients for baclofen treatment (Zhu et al., 2021b).

#### 5.2.2 Gabapentin

Gabapentin is a chemical analogue of GABA. It binds specifically to the α2δ subunit of intracranial voltage-regulated calcium channels and blocks neurotransmitter release at synapses (Cheng and Chiou, 2006). Gabapentin has been shown to help people with chronic neuropathic pain from central sensitization (Kimos et al., 2007). The pathophysiological causes of neuropathic pain and chronic cough are assumed to be comparable. Therefore, gabapentin is thought to potentially treat chronic refractory cough by inhibiting the sensitizing cough center. In the first clinical trial, 28 patients with persistent refractory cough associated with laryngeal sensory neuropathy were treated with gabapentin, with 68 percent of them experiencing cough alleviation (Lee and Woo, 2005). Gabapentin reduced symptoms in 5/6 individuals with persistent refractory cough of unclear etiology, according to a case report (Mintz and Lee, 2006). In a clinical trial, gabapentin was shown to significantly reduce symptoms in patients with chronic refractory cough after 8 weeks of treatment if increased gradually from 300 mg/day to 1800 mg/day (Ryan et al., 2012). A meta-analysis further supports that gabapentin is effective and safe in the treatment of chronic refractory cough (Shi et al., 2018). However, it has been studied that still 40% of patients with suspected refractory GERC do not respond to treatment with these two medications (Dong et al., 2019). Future study will focus on how to identify individuals with refractory GERC for gabapentin or baclofen treatment, increase efficacy, and reduce needless side effects.

#### 5.2.3 Acid suppression therapy

As with GERD, anti-reflux medications used to treat GERC include antacids, which reduce the acidity of the reflux, and pro-acids, which accelerate esophageal clearance or stomach emptying (Irwin, 2006). Therefore, antisecretory medicines like as proton pump inhibitors (PPIs) and H2 antagonists can be used alone or in combination with boosters to treat GERC, while the therapeutic value of PPIs is debatable (Kahrilas et al., 2013; Kahrilas et al., 2016; Morice et al., 2020). However, more than a third of GERC patients do not react to these treatments (Xu et al., 2016), and can be described as having “refractory GERC” (Lv and Qiu, 2015).

#### 5.2.4 Proton pump inhibitor (PPI)

PPIs are frequently used by patients with chronic cough, and in one study, 79% of patients with chronic cough experienced improvement in symptoms after PPI treatment (Poe and Kallay, 2003). However, a meta-analysis of adults with chronic cough found inadequate evidence to support PPI treatment (Chang et al., 2006). PPIs were not found to be more beneficial than placebo in treating chronic cough in adults in two other randomized controlled trials (Faruqi et al., 2011; Shaheen et al., 2011). As a result, the importance of acid suppression in the treatment of chronic cough of uncertain origin has been downplayed in the most recent expert panel guidelines (Gibson et al., 2016). The therapeutic role of PPI for GREC requires further and more comprehensive studies, such as how to screen specific GERC patients.

#### 5.2.5 H^+^/K^+^-ATPase blockers (P-CAB)

One innovative approach is the development of reversible H+/K + - atpase blockers, called P-CAB, which block the K+ exchange channel of the proton pump, leading to rapid, competitive, reversible inhibition of acid secretion (Hunt and Scarpignato, 2015). Mucosal healing in acid-associated disorders is strongly connected to the degree and duration of acid inhibition, as well as the length of treatment (Bell et al., 1992; Hunt, 1999; Katz et al., 2006). Given the limits of existing antisecretory medications, this new therapy has the potential to accomplish quick, effective, and long-lasting acid suppression, as well as to address the specific treatment demands of GERD patients (Hunt, 2005; Katz et al., 2006; Dickman et al., 2015). In the future, further evidence will be needed to evaluate the role of P-CAB in the treatment of GERC.

#### 5.2.6 Chinese herbal medicine

A clinical trial exploring herbal remedies for GERC has recently emerged, including patients without symptoms of gastrointestinal reflux (Bhang et al., 2020). In 2022, the trial was designed and demonstrated the potential of Ojeok-san plus Saengmak-san in alleviating the severity of cough and gastrointestinal symptoms in GERC patients with safe and successful feasibility results (Lyu et al., 2022). This gives new ideas for the future development of herbal drug combination in GERC.

#### 5.2.7 Mucosal protective drugs (alginate)

Increased mucosal permeability, also known as poor mucosal integrity, has been linked to the occurrence of GERD symptoms in several investigations (Kia et al., 2016). Formulations containing alginate show mucosal protective activity, forming a mechanical barrier by interacting with stomach acid to form a gel (Kwiatek et al., 2011). In a recent systematic review, 14 studies included found that alginate preparations had a significantly better symptomatic response than placebo or antacids (Leiman et al., 2017). A study has shown that in individuals with symptomatic GERD, alginate-antacid combination therapy has been demonstrated to be an effective and well-tolerated treatment for reducing reflux symptoms (Savarino and PaceF.SCarpignato, 2017). In an earlier study showed significant improvement in laryngeal signs in patients treated with alginate solution (McGlashan et al., 2009). And the effect of its use in GERC awaits verification in future studies.

#### 5.2.8 Prokinetic therapy

A study shows that dysmotility symptoms could be a therapeutic option for GERC (Kanemitsu et al., 2019). A meta-analysis of prokinetic treatment for suspected extraesophageal reflux, on the other hand, found inadequate evidence to support its use (Glicksman et al., 2014). Recent guidelines have recommended treatment options for GERD that do not respond to PPI therapy, and prokinetics was not among the treatment options reviewed by the panel (Yadlapati et al., 2018). Japanese guidelines do recommend the use of prokinetics in patients with refractory GERD after failure of PPIs (Iwakiri et al., 2022). Future therapeutic perspectives of prokinetic agents in Asians and their specific role in the treatment of GERC await further research.

#### 5.2.9 Anti-reflux surgery

Most surgical data on chronic cough are unplanned, retrospective, and based on small sample sizes (Mainie et al., 2005; Tutuian et al., 2006; Hoppo et al., 2013). Anti-reflux surgery is a high-risk therapy option for extraesophageal reflux because there is not enough evidence to back it up. Although anti-reflux surgery is an effective alternative treatment for GERC patients, it is only available to a small group of carefully selected patients (Schwameis et al., 2020). According to a recent study, surgical anti-reflux surgery is strongly advised to manage respiratory symptoms in GERD patients with proper patient selection (Tustumi et al., 2021).

#### 5.2.10 Breathing exercises

The benefits of breathing exercises can help GERD patients reduce reflux symptoms, improve quality of life, and reduce medication use (Casale et al., 2016; Qiu et al., 2020). Furthermore, it could be a potential treatment choice for PPI-refractory GERD patients and could help responder GERD patients reduce their annual PPI dose. As a result, GERD patients may benefit from combining conventional medication therapy with professional breathing exercises to achieve better therapeutic results (Eherer et al., 2012).

## 6 Conclusion and future direction

GERC is a subtype of GERD-related disease. Its pathogenesis is multifactorial and mainly involves reflux theory, reflex theory and esophageal dysmotility, while weak acid reflux and misaspiration may also be partly responsible. Diagnosis of GERC relies on careful medical history collection of typical cough symptoms, and records for the duration and number of symptom episodes. Besides, validated questionnaires such as FSSG and DMS can also be considered in diagnosis, but they have only been utilized infrequently in clinical practice. In addition, the presence of endoscopic lesions has a low diagnostic sensitivity, biopsy of mucosal thickness could be a useful marker awaiting further studies, and real-time MRI is gradually being developed for diagnosis. Because it can detect proximal reflux burden as well as acid and non-acid reflux, combined pH-MI is regarded the best diagnostic tool for extraesophageal reflux. The development of new impedance indicators (i.e., MNBI and PSPW) is expected to further improve the diagnostic yield of this technique in GERC patients. Taken together, most of the above diagnostic methods do not combine sensitivity, specificity, noninvasiveness and simplicity, so a noninvasive and sensitive diagnostic method is needed to diagnose GERC. Salivary amylase shows potentials, while future researches are still needed to develop noninvasive diagnostic tools.

PPIs are the backbone of GERD pharmacotherapy because they inhibit acid, which is still the most aggressive and destructive element in the disease’s pathophysiology. However, there are still some patients who show a partial or ineffective response to PPIs because there are other non-acid factors that can also cause reflux symptoms. PCAB is a faster-acting alternative acid inhibitor, but its clinical experience is limited. Prokinetic agents and reflux inhibitors have the ability to address the most critical pathophysiologic pathways that cause GERD, however clinical trial results are inconsistent. Standard treatment of GERC includes the use of proton pump inhibitors alone or in combination with prokinetic agents, and lifestyle changes. Patients who have a partial response to antisecretory medications alone may benefit from the use of mucosal protecting chemicals, which are typically used in conjunction with PPIs. There are also anti-reflux procedures and respiratory exercises awaiting further studies on their therapeutic role in GERC. Furthermore, refractory GERC is characterized as a chronic cough that has resisted normal therapy for at least 8 weeks but responds well to rigorous anti-reflux therapy. For refractory GERC, neuromodulators such as baclofen and gabapentin are being evaluated as therapy choices. Therefore, it may be a future direction to screen out specific GERC patients and make adjustments to the treatment plan in real time based on the treatment results.
