# Supplementary material for: GERD-related chronic cough: Possible mechanism, diagnosis and treatment
Source: Front Physiol. 2022 Oct 20;13:1005404. doi: 10.3389/fphys.2022.1005404 (PMC9630749; doi:10.3389/fphys.2022.1005404)
Supplement: Supplementary file 1 [file DataSheet1.docx]

**Table S1 Advantages and disadvantages of different diagnostic methods**

| Diagnostic methods | Advantages | Disadvantages |
| --- | --- | --- |
| Endoscopy | High relative safety, therapeutic promise and relative specificity | Low diagnostic rate and low patient tolerance |
| Biopsies | Decreasing additional costs, discomfort | Time-consuming, expensive, and less sensitive |
| Impedance pH detection | High sensitivity, high diagnosis rate and differentiation of reflux types | High cost and low patient tolerance |
| Symptom index (SI) and symptom association probability (SAP) | More statistical validity and high sensitivity | Low diagnostic rate |
| DeMeester score (DMS) | Assessing acid exposure time (AET) and improving diagnostic rates | High false negative rate |
| Mean nocturnal baseline impedance (MNBI) | High degree of differentiation | Low sensitivity |
| Post-reflux swallow-induced peristaltic wave (PSPW) | High sensitivity | Low accuracy rate |
| Mucosal impedance (MI) | High sensitivity and high accuracy rate | Time-consuming and low patient tolerance |
| Real-time magnetic resonance imaging (MRI) | High diagnosis rate and high patient tolerance | High cost |
| Proton pump inhibitor trial | Non-invasive and high patient tolerance | Low specificity |
| Narrow band imaging (NBI) | High sensitivity | Low accuracy rate |
| Salivary pepsin | Non-invasive and high patient tolerance | Low accuracy rate |
| The Frequency of Symptoms of Gastroesophageal Reflux Scale (FSSG) | Simple and high patient tolerance | Low accuracy rate |

**Figure S1 Diagnostic Flowchart**

**
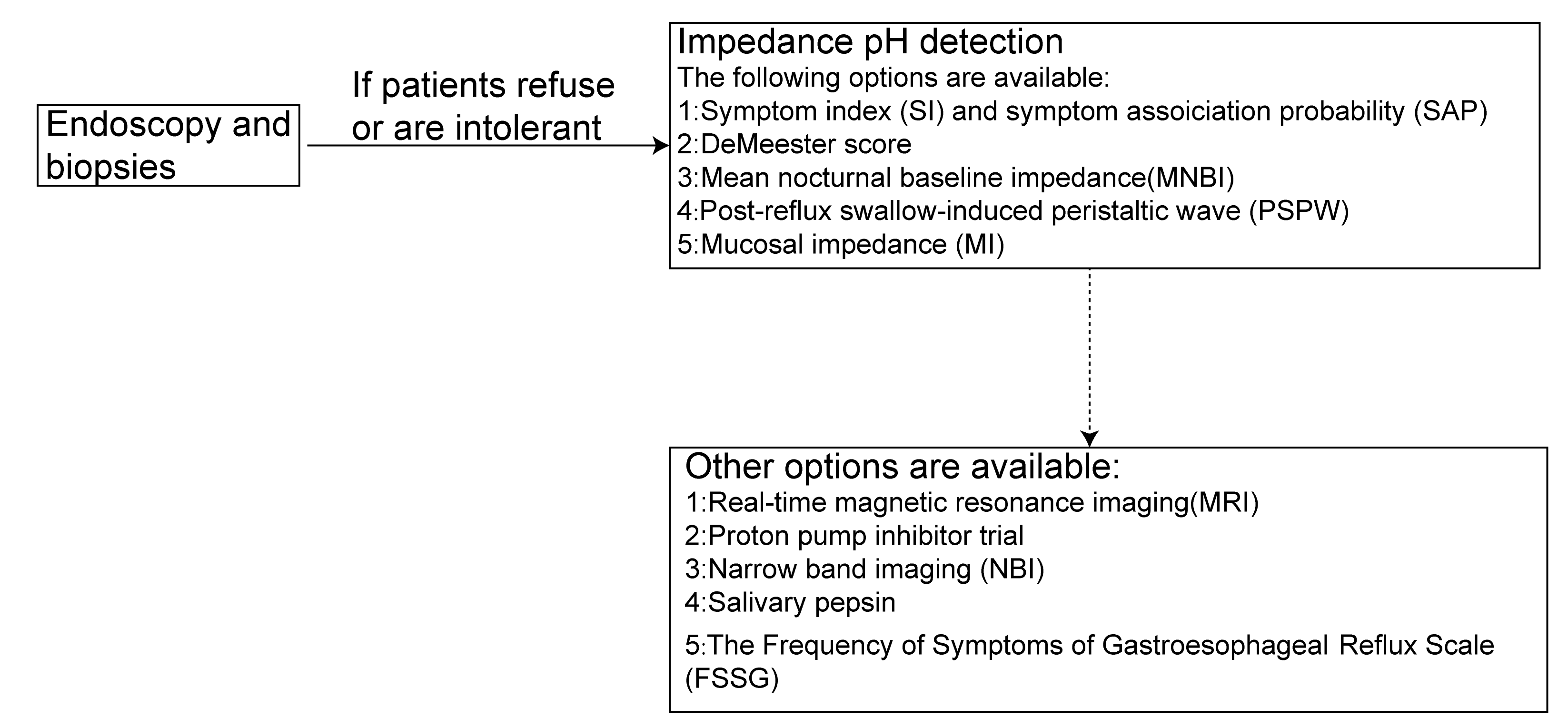
**

**Table S2 Mechanisms and advantages and disadvantages of different treatments**

| Treatment | Mechanism | Advantages | Disadvantages |
| --- | --- | --- | --- |
| Lifestyle Changes | Reducing risk factors | Low cost and high level of patient acceptance | Average treatment effect |
| Baclofen | Modulating transient LES relaxation mediated by the vagal reflex pathway through activation of GABA type-B receptors and cough suppressant activity | Effective in refractory GERC | Average treatment effect and many side effects |
| Gabapentin | Inhibiting the sensitizing cough center | High rate of relief for cough | Many side effects |
| PPI | Irreversible inhibition of the H+/K+-ATPase | Simple and high level of patient acceptance | Low rate of relief for cough and many side effects |
| H+/K+-ATPase blockers (P-CAB) | Reversible inhibition of the H+/K+-ATPase | Quick, effective, and long-lasting acid suppression | Lack of research in GERC |
| Chinese herbal medicine | Moisturizing the respiratory mucosa | High level of patient acceptance | Insufficient evidence, further research needed |
| Mucosal protective drugs (Alginate) | Mucosal protective activity | High level of patient acceptance | Lack of research in GERC |
| Prokinetic therapy | Inhibition of gastroesophageal motility | High level of patient acceptance | Lack of research in GERC |
| Anti-reflux surgery | Strengthening of the lower esophageal sphincter | Effective in carefully selected patients | High-risk and low level of patient acceptance |
| Breathing exercises | Reduced airway response | Simple and high level of patient acceptance | Average treatment effect |

**Figure S2 Flow chart of management**

**
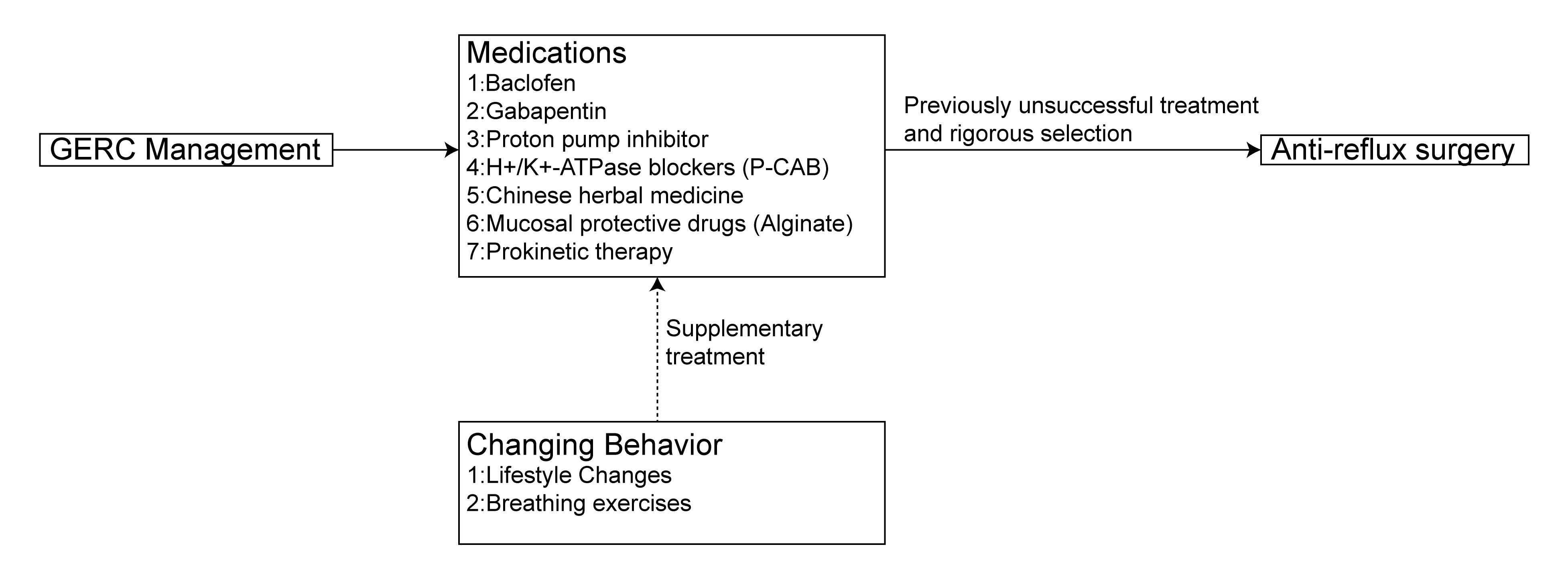
**
